# Mutational and acquired carbapenem resistance mechanisms in multidrug resistant *Pseudomonas aeruginosa* clinical isolates from Recife, Brazil

**DOI:** 10.1590/0074-02760150233

**Published:** 2015-12

**Authors:** Felipe Lira de Sá Cavalcanti, Cristina Rodríguez Mirones, Elena Román Paucar, Laura Álvarez Montes, Tereza Cristina Leal-Balbino, Marcia Maria Camargo de Morais, Luis Martínez-Martínez, Alain Antonio Ocampo-Sosa

**Affiliations:** 1 Universidade de Pernambuco, Instituto de Ciências Biológicas, Laboratório de Resistência Microbiana, Recife, PE, Brasil; 2 Universidade Federal de Pernambuco, Centro de Ciências Biológicas, Departamento de Genética, Recife, PE, Brasil; 3 Fundação Oswaldo Cruz, Centro de Pesquisas Aggeu Magalhães, Recife, PE, Brasil; 4 Instituto de Investigación Marqués de Valdecilla, Hospital Universitario Marqués de Valdecilla, Santander, Spain; 5 Universidad de Cantabria, Facultad de Medicina, Departamento de Biología Molecular, Santander, Spain

**Keywords:** Pseudomonas, *oprD* porins, β-lactamases, efflux, resistance

## Abstract

An investigation was carried out into the genetic mechanisms responsible for multidrug resistance in nine carbapenem-resistant *Pseudomonas aeruginosa*isolates from different hospitals in Recife, Brazil. Susceptibility to antimicrobial agents was determined by broth microdilution. Polymerase chain reaction (PCR) was employed to detect the presence of genes encoding β-lactamases, aminoglycoside-modifying enzymes (AMEs), 16S rRNA methylases, integron-related genes and OprD. Expression of genes coding for efflux pumps and AmpC cephalosporinase were assessed by quantitative PCR. The outer membrane proteins were separated by sodium dodecyl sulfate-polyacrylamide gel electrophoresis. The *bla*SPM-1*, bla*KPC-2 and *bla*GES-1 genes were detected in *P. aeruginosa*isolates in addition to different AME genes. The loss of OprD in nine isolates was mainly due to frameshift mutations, premature stop codons and point mutations. An association of loss of OprD with the overexpression of MexAB-OprM and MexXY-OprM was observed in most isolates. Hyper-production of AmpC was also observed in three isolates. Clonal relationship of the isolates was determined by repetitive element palindromic-PCR and multilocus sequence typing. Our results show that the loss of OprD along with overexpression of efflux pumps and β-lactamase production were responsible for the multidrug resistance in the isolates analysed.

The increasing prevalence of multidrug-resistant (MDR) and extensively drug-resistant (XDR) *Pseudomonas aeruginosa* isolates is severely compromising the selection of appropriate treatments for the infections caused by these organisms and is causing high morbidity and mortality (Poole 2011,Ocampo-Sosa et al. 2012). Although carbapenems remain effective for therapy of infections caused by *P. aeruginosa*, development of high resistance rates to carbapenems in this species has been reported worldwide (Poole 2011).

Carbapenem resistance is common in *P. aeruginosa*, especially in isolates from patients admitted to intensive care units (ICUs), where the selective pressure exerted by the use of antibiotics on bacterial populations is high. This is often related to the occurrence of mutations that inactivate the gene which codes for porin OprD, the specific portal of entry for carbapenems into this organism (Poole 2011). Mutational inactivation of*oprD* is the main mechanism of carbapenem resistance in the absence of acquired carbapenemases. Sequence analysis of the *oprD* gene usually reveals various routes of inactivation, including single nucleotide change resulting in premature stop codon, insertion or deletion resulting in frameshift, and disruption of protein by insertion sequences (Lee & Ko 2012).

*P. aeruginosa* may also acquire foreign genes encoding Ambler class A and class B β-lactamases that are able to hydrolyse carbapenems (Poole 2011). Although rarely identified, KPC-producing *P. aeruginosa* isolates have been reported, first in Colombia in 2007 and then in Puerto Rico, Trinidad and Tobago, the United States of America, and China (Villegas et al. 2007, Potron et al. 2015). In Brazil, the first case was reported in 2012 and involved two isolates recovered from a hospital located in Recife, state of Pernambuco (Jácome et al. 2012).

Metallo-β-lactamases (MBLs) hydrolyse carbapenems and other β-lactams (except monobactams) very efficiently and are not affected by the clinically available β-lactamase inhibitors (Potron et al. 2015). Among the MBLs, SPM-1 is an important determinant of MDR phenotype present in *P. aeruginosa* from Brazil and its dissemination has been caused by an epidemic (and endemic) *P. aeruginosa* ST 277 clone (Fonseca et al. 2010). This is evidence of its widespread distribution which has caused serious morbidity and mortality in hospital infections (Galetti et al. 2015). Further, overexpression of the MexAB-OprM and MexEF-OprN efflux system and chromosomal cephalosporinase AmpC can also lead to carbapenem resistance among *P. aeruginosa* clinical isolates when associated with other mechanisms (Poole 2011).

Aminoglycoside modification leading to antibiotic inactivation typically involves their phosphorylation, acetylation or adenylation by aminoglycoside-modifying enzymes (AMEs) (Poole 2011). A more recently discovered aminoglycoside resistance mechanism involves methylation of the 16S rRNA of the A site of the bacterial 30S ribosomal subunit, which interferes with antibiotic binding and so promotes high-level resistance to clinically relevant aminoglycosides like gentamicin, tobramycin, and amikacin in *P. aeruginosa* and other Gram-negative bacteria (Poole 2011).

A previous study conducted by our research team showed that resistance to β-lactam antibiotics (especially carbapenems in recent isolates of *P. aeruginosa*recovered from public hospitals in Recife) was not related to the spread of the Brazilian epidemic MBL (SPM-1) 48-1997A clone (Cavalcanti et al. 2012). Other resistance mechanisms should be present and responsible for the XDR phenotype observed, which needed further investigation. In Brazil, little is known about the synergistic effect of mutational and acquired mechanisms related to carbapenem resistance in MBL negative isolates, leading to this study.

We have analysed the alterations of OprD in a collection of carbapenem-resistant*P. aeruginosa* isolates from three public hospitals located in Recife. Sequence analysis of OprD was carried out to correlate inactivating mutations with the carbapenem resistance patterns observed. An investigation was also carried out into the presence of additional mechanisms involved in *P. aeruginosa*multidrug resistance, like β-lactamases, efflux pump overexpression, and enzymatic modification by aminoglycoside modifying enzymes and methylation of 16S ribosomal RNA by 16S rRNA methylases.

## SUBJECTS, MATERIALS AND METHODS

*Bacterial isolates* - Nine *P. aeruginosa* isolates were collected from different patients in three public hospitals in Recife (5 being from hospital A, 2 from hospital B, and 2 from hospital C) between 2008-2010. The criterion for selection was the presence of carbapenem resistance. Among the isolates, five were from patients hospitalised at ICUs, two were from patients in a cardiology unit, one was from an oncology unity, and another one was from an adult isolation facility. The most frequent sources of isolation were urine culture, and tracheal aspirate. One isolate that was susceptible to carbapenems (Ps 185) was included in all the experiments as a control. Species identification was performed by standard biochemical tests and confirmed by the VITEK-2 system and MALDI-TOF.

*Antimicrobial susceptibility testing and molecular typing* - The minimal inhibitory concentrations (MICs) of amikacin, gentamicin, tobramycin, arbekacin, ciprofloxacin, imipenem, meropenem, aztreonam, ceftazidime, and piperacillin-tazobactam were determined by broth microdilution. MIC breakpoints for all agents except arbekacin were those defined by EUCAST. Neither EUCAST nor CLSI have defined breakpoints for arbekacin, because of this and following previous recommendations (Zapor et al. 2010), we have considered the following criteria: ≤ 2, susceptible; ≥ 16, resistant.*Escherichia coli* ATCC 25922 and *P. aeruginosa*ATCC 27853 were employed as quality control strains. Clonal relatedness was evaluated through repetitive element palindromic-polymerase chain reaction (REP-PCR) in accordance with a previously described protocol (Vila et al. 1996). Multilocus sequence typing (MLST) was performed by using seven *P. aeruginosa* standard housekeeping genes (pubmlst.org/paeruginosa/). The assignment of allelic numbers and sequence type (ST) was determined after the comparison analysis.

*Phenotypic tests and molecular detection of gene coding for β-lactamases, integrases, aminoglycoside modifying enzyme, and 16S rRNA methylases* - The isolates were screened for carbapenemase production by the modified Hodge test (MHT) and for acquired MBLs production by the disk approximation test with 2-mercaptopropionic acid and the ethylenediamine tetraacetic acid-phenanthroline-imipenem microdilution test (Arakawa et al. 2000, Migliavacca et al. 2002, Amjad et al. 2011). Bacterial DNA was extracted by using the Instagene kit (BIO-RAD, USA) following the manufacturer’s recommendations. The presence of three MBL-encoding genes (*bla*_SPM_, *bla*_IMP_, and*bla*_VIM_) and *bla*_GES_ was investigated by specific PCR using previously described primers and conditions (Poirel et al. 2000, Cavalcanti et al. 2012). For detection of*bla*_KPC_, the primers 5’-TGTCACTGTATCGCCGTC-3’ (forward) and 5’-TTACTGCCCGTTGACGCC-3’ (reverse) were used (Endimiani et al. 2008). Screening for AMEs was performed by searching for genes that encode the acetyltransferases AAC(3)-Ia, AAC(3)-Ic, AAC(6’)-Ib-cr, AAC(6’)-IIa, the nucleotidyltranferases ANT(2’’)-Ia and ANT(4’)-IIb, the phosphotransferases APH (3’)-IIb and APH(3’)-VIa, and the 16S rRNA methylases ArmA, RmtA, RmtD, RmtC, RmtB, and NpmA, as previously described (Castanheira et al. 2008, Miró et al. 2012). The presence of integrons was determined by the amplification of *intI1*, *intI2*,*intI3* integrase genes, *qacE∆1*/*sul1*, and the use of specific primers to target the variable region of the integron (Mazel et al. 2000, Castanheira et al. 2004, Novais et al. 2006). Mapping of the region downstream the *sul1*gene in strain 922 was performed with primers Sul1-fw (Mazel et al. 2000), Sul1-outR (5’-GAATCGCGCCTTCGACA-3’), Orf494-intF (5’ACATGCTGTGGCTCGACG-3’), Orf494-intR*(5’-GCTTCCACGTACACGCC-3’), Orf494-int2R*(5’-CAGTGGACGCAGGCGG-3’), Orf494-divF (5’-GCAAGCGCGAACGACG-3’), RmtD-divR (5’-CGGGTGGGCAGATTGCT-3’), and RmtD-F (Castanheira et al. 2008). The PCR products were purified by means of the Wizard^®^ SV Gel and PCR Clean-Up System (Promega, USA) and sequenced through an external resource (Macrogen Inc, The Netherlands). The nucleotide and deduced protein sequences were analysed by on-line BLAST at GenBank dataset (National Center for Biotechnology Information).

*Plasmid analysis and transformation* - Plasmid DNA extracted by the PureYield™ Plasmid Miniprep System (Promega) was used for the transformation experiments with the aid of an electrocompetent *E. coli* TOP 10 as recipient cell. Transformants were selected on Luria-Bertani agar plates with 4 µg/mL ceftazidime. MICs of transformants, donor, and recipient strains were determined by Etest and interpreted according to the EUCAST guidelines. Plasmid replicons were typed by employing the PCR-based replicon typing method (Carattoli et al. 2005). The estimation of the plasmid size was performed by S1 nuclease treatment of plasmid DNA followed by pulsed field gel electrophoresis (PFGE).

*Sequencing of the oprD gene and analysis of the outer membrane proteins (OMPs)* - The presence of inactivating mutations in*oprD* was investigated in all the isolates by PCR amplification and sequencing, as described earlier (Ocampo-Sosa et al. 2012). The analysis of the OMPs was performed by following a protocol described elsewhere (Mushtaq et al. 2004). The OMPs were run on standard 12% sodium dodecyl sulphate-polyacrylamide gel electrophoresis (SDS-PAGE) and stained with Coomassie Blue. The OprD profiles from clinical isolates were compared with those of the reference strain PAO1 and PAO45 (Ocampo-Sosa et al. 2012).

*Quantitative real-time PCR (qRT-PCR)* - The expression of*ampC*, *mexB*, *mexD*,*mexF*, and *mexY* genes was determined by qRT-PCR. Total RNA was extracted using the SV Total RNA Isolation System (Promega). For the cDNA synthesis, 1 µg of RNA was treated with a DNA-free Kit (Applied Biosystems, USA) to eliminate the DNA contamination. Reverse transcription was performed in accordance with the protocol for the use of iScript cDNA Synthesis Kit (BIO-RAD). The quantification of transcripts was carried out by means of SYBR Premix Ex Taq (Takara). The relative gene expression was calculated by employing the 2^-∆∆Ct^ method. Expression of the constitutive gene*proC* was assessed in parallel to standardise the transcriptional levels of the target genes. Strains were considered positive for*ampC*, *mexD*, *mexF*, or*mexY* overexpression when the corresponding mRNA level was at least 10-fold higher than that of PAO1, negative if lower than five-fold, and borderline if between five-10-fold (Cabot et al. 2011). Strains were considered (i) positive for *mexB*overexpression when the corresponding mRNA level was at least three-fold higher than that of PAO1, (ii) negative if lower than two-fold, and (ii) borderline if between two-three-fold (Cabot et al. 2011). All the experiments were performed in technical and biological triplicates.

*Ethics* - This work was approved by the Ethical Committee of the State University of Pernambuco, Brazil (reference 265.604).

## RESULTS

*Antimicrobial susceptibility of the strains* - The antimicrobial susceptibility analysis revealed that most of the isolates were resistant to all the antibiotics tested (Table). The Ps 185 isolate showed susceptibility to imipenem and meropenem. On the other hand, the Ps 609 was susceptible to amikacin, gentamicin, tobramicin, ciprofloxacin, ceftazidime, and piperacillin-tazobactam.

**TABLE:** Mechanisms of resistance to selected antimicrobial agents detected among the *Pseudomonas aeruginosa* isolates

Isolate	ST	REP-type	MIC (µg/mL)										Determinant of antimicrobial resistance	Alteration/ effect on OprD expression
			AK	GM	TOB	ABK	CIP	IMI	MER	CAZ	ATM	TZP		
901	1560	C	64	> 64	> 64	32	32	32	128	> 128	32	64	*bla*_GES-1_;*aac(6’)-Ib*; *catB3*;*arr-4*; AB+; XY+; AmpC+	_nt 561_ DC/ premature stop codon and porin loss
902	1419	D	> 128	> 64	> 64	8	32	64	64	> 128	64	128	*bla*_GES-1_;*aac(6’)-Ib*; *ant(2’)-Ia*;*aph(3’)-VIa*; *catB3*;*arr-4*; AB+; XY+	_nt 1251_ G→A/ premature stop codon and porin loss
920	1126	D	> 128	> 64	> 64	8	32	64	64	> 128	32	64	*bla*_GES-1_;*aac(6’)-Ib*; *ant(2’)-Ia*;*aph(3’)-VIa*; *catB3*;*arr-4*; AB+; XY+	_nt 1251_ G→A/ premature stop codon and porin loss
922	277	E	> 128	> 64	> 64	128	> 32	> 256	> 256	> 128	16	128	*bla*_SPM-1_;*bla*_OXA-10_;*aac(6’)-Ib*; *qacED1*;*sul1*; *rmtD*; XY+	_nt 38_ DTG/ premature stop codon and porin loss
185	235	A	128	> 64	> 64	64	16	0.5	2	16	32	32	*ant(2’)-Ia*;*ant(3’’)-Ia*; *qacED1*;*sul1*; AB+; XY+	Silent mutations/ full length OprD
609	446	B	4	2	0.5	2	0.2	16	16	8	16	8	AB+	_nt 826_ CACC _nt 827_/ premature stop codon and porin loss
347	235	A	128	> 64	> 64	64	16	> 256	> 256	> 128	> 128	256	*bla*_GES-1_;*bla*_KPC-2_; *ant(2’)-Ia*;*ant(3’’)-Ia*; *qacED1*;*sul*1; AB+; XY+	_nt 536_ GGGCC _nt 537_/ premature stop codon and porin loss
176	235	A	128	> 64	> 64	64	16	> 256	> 256	> 128	> 128	256	*bla*_GES-1_;*bla*_KPC-2_; *ant(2’)-Ia*;*ant(3’’)-Ia*; *qacED1*;*sul*1; AB+; XY+	_nt 536_ GGGCC _nt 537_/ premature stop codon and porin loss
4269	244	F	16	> 64	> 64	4	32	128	256	64	64	256	*aph(3’)-VIa*; AB+; XY+; AmpC+	_nt 1205_ C _nt 1206_/ frameshift mutation and stop codon 92 bp downstream of the canonical TAA (larger, nonfunctional protein)
6553	244	F	> 128	> 64	64	2	32	256	> 256	> 128	> 128	256	*bla*_KPC-2_;*aph(3’)-VIa*; AB+; XY+; AmpC+	_nt 1205_ C _nt 1206_/ frameshift mutation and stop codon 92 bp downstream of the canonical TAA (larger, nonfunctional protein)

AB+, XY+, and AmpC+ designed MexAB, MexXY, and AmpC overexpression, respectively. ABK: arbekacin; AK: amikacin; ATM: aztreonam; CAZ: ceftazidime; CIP: ciprofloxacin; GM: gentamicin; IMI: imipenem; MER: meropenem; MIC: minimal inhibitory concentration; REP: repetitive element palindromic; ST: sequence type; TOB: tobramycin; TZP: piperacilin/tazobactam.

*β-lactamases*, *integrons, aminoglycoside modifying enzymes, and detection of 16S rRNA methylases* - Four isolates (Ps 176, Ps 347, Ps 922, and Ps 6553) showed presumptive carbapenemase production by the MHT. Nine out of 10 isolates were found to be negative for MBL production by both the phenotypic and genotypic test (survey of *bla*_SPM_,*bla*_IMP_, and *bla*_VIM_genes). Only one isolate (Ps 922) was positive for MBL and harboured the*bla*_SPM-1_ gene. Three isolates were positive for the presence of *bla*_KPC-2_ and five had*bla*_GES-1_ (Table).

Nine out of 10 isolates possessed Class 1 integrons, which was confirmed by amplification of the *intl1* integrase gene and*qacEΔ1* and *sul1* that are present in the conserved segment (3’-CS) of this genetic element. None of the isolates of this study harboured Classes 2 or 3 integrons.

Sequencing showed that strains Ps 901, Ps 902, and Ps 920 displayed two distinct Class 1 integrons, one of them showed a variable region of 2,350 bp harbouring the*bla*_*GES-1*_, with the gene cassette arrangement [*intI1*_*bla*_*GES-1*__*aac(6’)-Ib*], showing a 97% identity with that of *P. aeruginosa* strain CB1 (GenBank accession: KM210290.1). The second Class 1 integron found in these strains showed a variable region 2,098 bp and contained four gene cassettes namely,*intI1*, *aac (6’)-Ib*, *catB3*, and *arr-4*, which shared a 98% identity with a Class 1 integron previously identified in *P. aeruginosa* strain PS1111 (GenBank accession: EF660562.1).

Strains Ps 176 and Ps 347 were also found to harbour two different Class 1 integrons (regions variables of 2,250 bp and 2,125 bp, respectively). In one of them, only the *intI1* and *bla*_GES-1_ genes could be amplified in the conditions applied. The other integron had the cassette arrays [*intI1_ant(3’’)-Ia_orfD_qacED1/sul1*] and shared a 98% identity with that of *P. aeruginosa* strain MMA83 (GenBank accession: HF546976.1). Strain Ps 185 also showed this integron structure.

In spite of being positive for the Class 1 integrases, no integron arrays could be amplified in strains Ps 4269 and Ps 6553. On the other hand, the location of the*bla*_*KPC-2*_ in strains Ps 176, Ps 347, and Ps 6553 could not be determined in the conditions used in this study. Similarly, the genetic environment of *bla*_*SPM-1*_ in strain Ps 922 could not be mapped with the primers and conditions employed. Nevertheless, a Class 1 integron with a region variable of 5,210 bp was found with the gene cassettes arrangement [*intI1*_*aac(6’)-Ib_bla*_*OXA-10*_ *_ISPa21_qacED/sul1*] . This integron array showed a 96% homology with that found before in *P. aeruginosa* PA095 (GenBank accession: DQ914960.2).

The search for AMEs genes revealed that five isolates carried the ANT(2’)-Ia enzyme and four carried the APH(3’)-VIa phosphotransferase (Table). The *rmtD* gene, coding for a 16S rRNA methylase, was found in strain Ps 922, which displayed a high MIC of arbekacin (128 µg/mL). Mapping of a 3,852 bp region downstream of the *sul1* gene showed that *rmtD* had the same location as previously described in*P. aeruginosa* PA095 (Doi et al. 2007).

*Molecular typing* - The REP-PCR typing revealed the presence of six different profiles namely: A (3 isolates), B (1 isolate), C (1 isolate), D (2 isolates), E (1 isolate), and F (2 isolates). MLST analysis showed that the isolates had different STs (Table), with three isolates (including 2 producing KPC and GES) belonging to ST 235 (REP-A profile). Other STs were also found: ST 446 (1 isolate), ST 1560 (1 isolate); ST 1419 and 1126 (2 isolates with REP-D profile); ST 277 (1 isolate) and ST 244 (2 isolates with REP-F profile).

*Plasmid typing and bacterial transformation* - Three different replicons were identified among the *P. aeruginosa* isolates: U (Ps 176 and Ps 347), P (Ps 901, Ps 902, Ps 920, Ps 922, Ps 4269, and Ps 6553), and FIA (Ps 902, Ps 920, Ps 4269, and Ps 6553).

Plasmid DNA from two isolates producing both*bla*_*KPC-2*_ and*bla*_*GES-1*_ (Ps 176 and Ps 347), was successfully used for bacterial transformation. Two transformant colonies of each strain were selected. The *bla*_KPC-2_ gene was present in all of them, while *bla*_GES-1_ was only transferred to the two transformants of Ps 347. An increase in the MICs of carbapenems was also observed for transformants from 0.19-1.5 µg/mL (isolate 347) and from 0.19-3 µg/mL (isolate 176) for imipenem, from 0.032 up to 0.75 µg/mL (isolate 347) and from 0.032 up to 2 µg/mL (isolate 176) for meropenem, from 0.032-1.5 µg/mL (isolate 347) and from 0.032 up to 8 µg/mL (isolate 176) for ertapenem, when compared with the recipient cell. The plasmid typing revealed that the plasmids that were transferred all belonged to IncU type.

All the plasmids that were visualised in eight out of 10 strains by PFGE of S1 nuclease-digested DNA, fell into an approximate range of 40-120 kb: Ps 176 (45 kb, 50 kb), Ps 347 (40 kb, 75 kb), Ps 901 (60 kb, 100 kb), Ps 902 (48 kb, 100 kb), Ps 922 (120 kb), Ps 4269, and Ps 6553 (40 kb, 120 kb). No plasmids were visualised in strains Ps 185 and Ps 609 under the experimental conditions used in this study. In Ps 920, despite the detection of two Inc groups (P and FIA), only one plasmid was detected (100 kb).

*Analysis of OprD expression and mutations in the oprD gene* - Sequencing analysis and comparison with the PA01 *oprD* gene showed the presence of mutations that led to a loss of porin and resistance to carbapenems in nine isolates (Table). Seven isolates showed point mutation, deletions, substitution or an addition of one-five nucleotides leading to premature stop codons. The other two isolates displayed frameshift mutations by insertion of 1 bp (Table). Analysis of outer membrane protein by SDS-PAGE confirmed the absence of the porin (~48 kDa) in these same isolates and its presence in a single carbapenem-susceptible isolate (Ps 185) used as a control, which had silent mutations in its *oprD* sequence (Figure).

**Figure:**
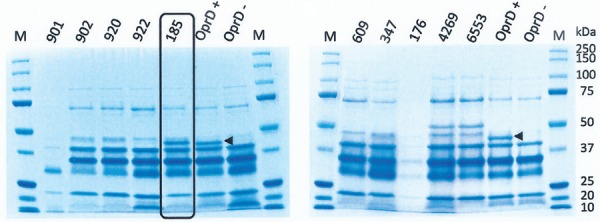
Sodium dodecyl sulphate-polyacrylamide gel electrophoresis of*Pseudomonas aeruginosa* outer membrane proteins, highlighting the strain susceptible to carbapenems and with basal expression of OprD. The arrowheads show the band corresponding to OprD. M: molecular marker (Precision Plus Protein Standards-BIO-RAD); OprD-: deficient mutant PAO45; OprD+: PA01.

*Overexpression of efflux systems and ampC* - There was an investigation of the relationship of carbapenem resistance with the efflux system and *ampC* overexpression (Table). The isolates overexpressed one or more genes of the efflux systems or *ampC*. Overexpression of the *mexB* (from 2 to 8-fold) and *mexY* (from 5 to 33-fold) was found among the isolates. However, overexpression of *mexD* and *mexF*was not observed. Three isolates that overexpressed MexAB-OprM and MexXY-OprM simultaneously hyperexpressed the *ampC* gene (from 17 to 27.5-fold compared with PA01). One isolate (Ps 609) that was susceptible to the aminoglycosides (amikacin, gentamicin, and tobramycin) did not overexpress MexXY-OprM (Table).

## DISCUSSION

The carbapenem resistance in *P. aeruginosa* in Brazil is still often associated with the production of class B β-lactamases; however isolates producing class A carbapenemase (KPC) were also found in this work. Although KPC enzymes are almost entirely attributed to enterobacteria, especially *Klebsiella pneumoniae* and *E. coli*, these β-lactamases have recently been observed in *Pseudomonas* spp in America (Villegas et al. 2007). KPC-2-producing*K. pneumoniae* have often been found in Brazil and recently, for the first time, in a *Pseudomonas putida* isolate recovered from the same hospital in Recife as the two KPC positive *P. aeruginosa*isolates used in this study (Almeida et al. 2012).

Bacterial transformation suggested that both *bla*_GES-1_ and*bla*_KPC-2_ were contained in IncU-type plasmids, however hybridisation experiments to confirm this (data not shown) failed to give conclusive results, so it is possible that one of the two plasmids belonged to an Inc group not investigated or was not transferred to the transformants cells. In a recent report, two plasmids carrying *bla*_KPC-2_ in*P. aeruginosa* isolates in Colombia were completely sequenced and one of them displayed a replicase gene of IncU. The isolates also belong to ST 235, which provides evidence that these isolates are somehow related to the Recife isolates and have a common origin (Naas et al. 2013). The single SPM-1 positive isolate (Ps 922) belonged to ST 277 and this finding is in agreement with that of a previous work which carried out the MLST typing of 50 isolates of SPM-1-producing *P. aeruginosa*, collected from 11 different cities in Brazil (Silva et al. 2011). These authors found that all but one strain belonged to ST 277, which confirmed the similarity of these isolates in the country (Silva et al. 2011). Despite the small number of isolates analysed, the MLST results highlight the variability found, suggesting the existence of different ST circulating in carbapenem-resistant *P. aeruginosa* isolates in Recife. However, the limited amount of sampling prevents any inference from being made about the possible epidemiological effects of this finding. In this study, there was an investigation into the occurrence of different AMEs and 16S rRNA methylases genes, and their correlation with resistance to aminoglycosides. The presence *aph(3’)-VIa* and*ant(2”)-Ia* seemed to be a significant cause of the high resistance to amikacin, gentamicin, and tobramycin observed in most isolates. This finding is consistent with a previous report, which demonstrated that both of these modifying enzymes were most often found in isolates of *P. aeruginosa* in Iran (Vaziri et al. 2011).

RmtD is the fifth 16S rRNA methylase identified in Gram-negatives, the second in*P. aeruginosa* (after RmtA), and the only one in this species that has so far been identified in Brazil (Doi et al. 2007). In our case, the *rmtD* gene was located on an integron that is very similar to the one previously described in a *P. aeruginosa* strain isolated in Brazil, that also contained*bla*_*SPM-1*_ (Doi et al. 2007). Additional studies that include more isolates are needed to know whether there is a close association between these two resistant determinants among multiresistant *P. aeruginosa* clinical strains in Brazil, which may represent a real threat to public health.

The different patterns found by molecular typing show variability between the isolates and the carbapenem resistance found in the *P. aeruginosa*isolates circulating in the hospitals evaluated was caused by association of different resistance mechanisms and could not just be attributed to a SPM-1 activity, as previously seen (Cavalcanti et al. 2012).

Inactivating mutations, found in the *oprD* sequences (whether or not associated with the production of carbapenemases), clearly demonstrated that the resistance to carbapenems (mainly imipenem) in the isolates of the current work, was due to a loss of porin, often the most prevalent mechanism in *P. aeruginosa*(Poole 2011). This could be observed by a significant reduction in the MIC of the transformants for imipenem when compared with the wild-types strains with the *oprD* mutations (Table). All of the carbapenem-resistant isolates overexpressed at least one gene among the four major efflux systems and*ampC*. Overexpression of MexAB-OprM and MexXY-OprM were the most prevalent, followed by AmpC. Despite showing *mexB* overexpression, the isolate Ps 185 was susceptible to carbapenems, which corroborates the belief that expression of efflux pumps in the absence of other mechanisms such as carbapenemase production or porin loss may not be sufficient to raise the MICs above the clinical breakpoints. In a clinical setting, overexpression of efflux pumps and defects in OprD function, synergistically increase the MIC of carbapenems compared with when either mechanism is used alone (Li et al. 2012). It is known that *mexXY* expression may be induced by fluoroquinolones and aminoglycosides (Xavier et al. 2010). This fact could in part explain why all the isolates that overexpressed *mexY*, showed high MICs values for amikacin, gentamicin, tobramycin, and ciprofloxacin.

This study demonstrates that OprD loss and KPC production were the main mechanisms for the carbapenem resistance in MBL-negative *P. aeruginosa*isolates from Recife. The presence of multiple resistance mechanisms in a single isolate, as observed in this work, further restricts the therapeutic options available for empirical treatment and the chance of achieving clinical success in infections caused by *P. aeruginosa*.
